# Religiosity and Psychotic Ideation in Stable Schizophrenia: A Role for Empathic Perspective-Taking

**DOI:** 10.3390/bs10020053

**Published:** 2020-02-05

**Authors:** Rosó Duñó, Joan Carles Oliva, Adolf Tobeña, Diego Palao, Javier Labad

**Affiliations:** 1 Servei de Salut Mental, Parc Taulí Hospital Universitari, CIBERSAM, Universitat Autònoma de Barcelona, 08193 Bellaterra, Barcelona, Spain; dpalao@tauli.cat (D.P.); jlabad@tauli.cat (J.L.); 2 Unitat de Neurociència Traslacional, Parc Taulí Hospital Universitari, Universitat Autònoma de Barcelona, 08193 Bellaterra, Barcelona, Spain; 3 Unitat d’Estadística i Avaluació, Institut d’Investigació i Innovació Parc Taulí, Universitat Autònoma de Barcelona, 08193 Bellaterra, Barcelona, Spain; jcoliva@tauli.cat; 4 Institut de Neurociències, Departament de Psiquiatria i Medicina Legal (Facultat de Medicina-Bellaterra Campus), Universitat Autònoma de Barcelona, 08193 Bellaterra, Barcelona, Spain

**Keywords:** empathy, perspective-taking, mentalizing, religiosity, schizophrenia

## Abstract

The relationship between religiosity and different components of empathy was explored in schizophrenia patients. A total of 81 stable schizophrenia patients and 95 controls from the nearby community completed self-reported questionnaires assessing religiosity and empathy (through the Interpersonal Reactivity Index, IRI). Patients with schizophrenia showed higher religiousness than controls and they presented less perspective-taking and empathic concern but increased personal distress in IRI scores. Regression analyses unveiled an association between religiosity and perspective-taking in schizophrenics after adjusting for age, gender, and psychotic symptoms. In conclusion, religiosity in patients with schizophrenia may be linked to variations in perspective- taking as a component of empathy.

## 1. Introduction

Religiousness plays a salient role in the everyday lives of schizophrenia patients, although their involvement in public religious activities is lower than that of the general population [1,2,3,4]. Research on religiosity and schizophrenia has mainly focused on delusions with religious content, thus linking transcendence ideation to psychopathology [5]. The prevalence of delusions and hallucinations with religious content varies among cultures and historical periods, although they are becoming less common in Western countries due to the decline of religious practices [6,7]. Typically, religious delusion topics are persecutory (often devil or demons), grandiose (believing to be God, Jesus, or an angel), or related to worries of condemnation (unpardonable sins) [5,8]. 

Religious tendencies rest on beliefs or experiences regarding supernatural agents and thoughts/hopes about transcendence [9]. There are cognitive processes that underlie the propensity toward religious thinking, particularly misguided agency attributions (assigning humanlike traits to nonhuman beings or objects and presuming ’intentions’ or purposes of external events), as well as a wide variety of mentalizing processes [10,11,12]. Mentalizing is a prerequisite for the appearance of the complex representations typically ascribed to Gods and other kinds of supernatural agents found in most religious beliefs [13]. Religious people tend to perceive God as an actual person rather than a fictional image, and several studies have shown that praying activates brain regions that are responsible for active interpersonal interactions [12,14,15,16]. Variations in religious propensities have been linked to the routine operation of social cognition processes involved in awareness, encoding, retrieval, and dealing with information about other people [13,17,18]. Empathy is an important ingredient of social interactions and comprises related but distinct affective and cognitive processes through which ‘perceivers’ (individuals focusing on another person’s internal states) relate to ‘targets’ (individuals who are the focus of perceivers’ attention) [19]. 

Schizophrenic disorders typically appear as a mixture of positive symptoms (disorganized behavior, delusions, and perceptual aberrations such as auditory hallucinations) and negative symptoms (a cluster of deficits that affect motivation—asociality, avolition, anhedonia—and emotional expressivity—alogia and flat affect) [20], accompanied by altered cognitive deficits, affecting both social and non-social areas. Patients with schizophrenia show worse performance than healthy controls in multiple social cognitive domains, including social perception, emotion perception and processing, and mentalizing abilities [21]. 

Empathy as a cognitive domain is clearly impaired in schizophrenia [22]. Research on empathy has focused in two general domains: *Cognitive* and *affective empathy*. Historically, research on empathy has mainly used a self-report measure called the Interpersonal Reactivity Index (IRI) [23], which includes two subscales which are often described as measures of *cognitive empathy*, perspective-taking and fantasy, and another two subscales, empathy concern and personal distress, which are often described as measures of *affective empathy* [24,25]. 

Cognitive empathy, also referred to as mentalizing, is defined as a set of reflective processes that includes taking the ‘perspective’ of others, understanding the emotional state of others, distinguishing another’s feelings from one’s own, and being able to integrate this information with social knowledge to adaptively guide interpersonal behavior [25,26]. Affective empathy refers to a set of relatively automatic processes through which perceived social cues trigger an emotional response in oneself that is shared with an observed person [25]. The capacity to accurately and adaptively empathize is believed to involve coordinated interaction between several of these processes [19]. Most studies have indicated that patients with schizophrenia have similar profiles to those of healthy controls on IRI subscales [26], although other research has found lower scores on empathic concern and elevated personal distress [27,28,29,30]. 

Although there are no data relating variations in empathy with religiousness, measures in schizophrenia and preliminary findings in schizotypic and autistic samples have suggested a plausible link [13,17,31,32]. Wlodarski and Pearce [13] found that schizotypal traits were associated with agency, mentalizing, and perceptual misattributions, along with peculiar religious behavior. Additionally, Willard and Norenzayan [32] demonstrated that some variations in agency detection and ability to mentalize led schizotypal individuals to more extreme forms of religious beliefs and behavior. Finally, in patients on the autistic spectrum, they found that their mentalizing deficits were associated with reduced belief in God [17,32].

In this exploratory study, we wanted to discern the existence of potential differences between stable schizophrenia patients with comparable controls, both in self-report measures of religiousness and in various empathy components, as measured by IRI scales. We expected first that patients with schizophrenia would show greater religiousness and lower empathy scores than people from the general population. As a second aim, we wanted to explore potential relationships between IRI empathy components and religiousness. We did this through different steps, using total religiousness scores first and three distinctive ingredients of religiosity afterwards (current beliefs, childhood religiousness, and moral perspective), while adjusting for age and gender. We expected, finally, to be able to disentangle plausible links between ingredients of empathy and positive and negative symptoms in schizophrenia. In this way, we expected to unveil association trends that might contribute to shedding light on peculiarities of schizophrenic ideation patterns that may give way to their empathic inadequacies. 

## 2. Method

Eighty-one patients (from 18 to 60 years) with schizophrenia (diagnosed according the DSM-IV RT criteria [33]) were studied and compared to a control group of ninety-five individuals (from 18 to 60 years) recruited from a nearby community (Vallès Oriental, Barcelona region, Spain). This control group was recruited through advertisements placed at different sites. They completed the requested questionnaires anonymously (see below). All patients included in this cross-sectional study were clinically stable [34] and received a survey administered during the years 2013–2014 by consecutive sampling at the Day Hospital, Psychiatry Rehabilitation Center, Parc Taulí Hospital (Sabadell, Barcelona, Spain). The exclusion criteria were: Not giving consent to participate in the study, visual or auditory disabilities limiting test application, neurological diseases or another chronic acute conditions that could interfere with cognitive performance, and additional DSM-IV RT diagnoses on Axis I/II [33]. 

### 2.1. Assessment 

All participants grew up with different degrees of exposure to the Catholic religion within the main Spanish culture. Their degree of religiousness was measured with a 25 item questionnaire developed by Kapogiannis et al. [9], which uses a Likert scale from 1 (disagreement with) to 7 (agreement with) for rating each item. The scale explores three different dimensions: Current religious beliefs, childhood religious practice, and moral relativism (pragmatism: Beliefs in distinctive moral obligations adjustable to the circumstances). This questionnaire assumes that religiosity profiles are based on clusters of traits that have influence over cognitive and affective strategies and behaviors over time, and it has been previously used in Spanish populations [35,36]. 

Empathy was assessed with the Interpersonal Reactivity Index (IRI) [23], Spanish-adapted version [37,38]. The IRI comprises 28 items rated on a Likert-type scale that ranges from 1 (‘not at all like me’) to 5 (‘very much like me’). It measures cognitive and emotional empathy across four subscales, but our analyses focused on three IRI scales: *Cognitive components*—Perspective-Taking (mentalizing: Tendency to adopt others’ points of view), *Affective components*—Empathic Concern (tendency to internally generate affective concern for others), and Personal Distress (tendency to feel distressed in the presence of others’ emotional distress). The IRI has been used extensively on healthy subjects [39,40], as well as on individuals with different psychiatric disorders, including schizophrenia [41,42]. 

The clinical severity of psychopathological symptoms (positive symptoms, negative symptoms, and general psychopathology) was assessed with the Positive and Negative Syndrome Scale (PANSS) [43]. For the purposes of the present study, individual symptoms were displayed and scored as well. This scale was applied by an expert staff psychiatrist of the Day Hospital (R.D.), who was trained in scale administration and was blind to religiosity and empathy measures.

The study was performed in accordance with the Declaration of Helsinki and was approved by the ethics committee of the Parc Taulí Hospital.

### 2.2. Statistical Analysis

Data were analyzed using SPSS 21.0 (SPSS, Inc., Chicago, IL, USA). Histograms, Q-Q plots, and Shapiro–Wilk tests were used for assessing the distribution of continuous variables and testing for normality.

#### 2.2.1. Univariate Analysis

Inferential statistics were calculated by χ^2^ and *t*-tests as appropriate. *p*-Values < 0.05 were considered statistically significant. The first hypothesis (comparison of religiousness and IRI measures between groups) was tested with *t*-tests.

#### 2.2.2. Multiple Linear Regression Analyses Considering Total Religiousness Score

Multiple linear regression analyses were performed in order to discern possible relationships between religiousness and IRI measures while adjusting for age and gender (as well as positive and negative symptoms in patients with schizophrenia). A stratified analysis by diagnosis was conducted; thus, we explored the associations between religiousness and IRI measures independently in schizophrenia patients and controls. In these multiple linear regression analyses, religiosity scores were considered the dependent variable. This variable followed a normal distribution (Shapiro–Wilk test was non-significant). Independent variables were entered into the equation in different steps. Age and gender were entered in a first step with the entry procedure. In those analyses with schizophrenia patients, positive and negative symptoms on the PANSS scale were also included with an entry procedure in a second step. Finally, the empathy dimensions of the IRI test were included in a last step by means of a stepwise procedure. Although we decided to select relevant empathy dimensions with a stepwise procedure, previous covariates (age, gender, and positive and negative symptoms) were included with the entry procedure to ensure that the final equations would be adjusted for these covariates.

#### 2.2.3. Multiple Linear Regression Analyses Considering Religiousness Dimensions

We conducted additional multiple regression analyses with the aim of exploring the relationship between empathy and different religiousness dimensions. Separate multiple linear regression analyses were conducted for each dimension, which was considered the dependent variable. Each of these religiousness dimensions was defined by its scores in the three subscales of the Kapogiannis scale: Current religious beliefs, childhood religious practice, and moral relativism. We considered the same covariates and selection procedures used in the main multiple linear regression analysis dealing with overall religiousness. 

## 3. Results

### 3.1. Univariate Analyses

Socio-demographic data and clinical characteristics including religiousness and IRI measures of schizophrenia patients and healthy controls are described in Table 1. In comparison to controls, patients with schizophrenia scored significantly higher in religiousness (overall score) and religion beliefs. In terms of empathy scores, patients with schizophrenia reported lower perspective taking and empathic concern but increased personal distress.

### 3.2. Multiple Linear Regression Analyses in Schizophrenia Patients

#### 3.2.1. Overall Religiousness

Regression analyses showed a significant positive association between religiousness (overall score) and perspective-taking, which was maintained after adjusting for covariates (Table 2). As can be seen in this table, the positive and negative PANSS scores were the variables more clearly related with religiousness (total score). In the last step (Model 3), of all IRI dimensions, only perspective-taking entered the final equation. The addition of perspective-taking increased the R^2^ of the model from 0.28 (Model 2) to 0.33 (Model 3), which means that inclusion of this scale in the multiple linear regression analysis only accounted for 5% of the data variability.

#### 3.2.2. Religiousness Dimensions

Further analyses for each of the three Kapogiannis subscales are shown in Tables S1–S3 of the Supplementary Results. 

In relation to current religious beliefs (Table S1), female gender was significantly associated with this religiousness dimension (β = 0.28; *p* = 0.012). Positive symptoms were also significantly associated with this dimension (β = 0.40; *p* = 0.002), whereas negative symptoms were inversely associated with this dimension (β = −0.43; *p* = 0.001). None of the IRI dimensions entered the equation by means of a stepwise procedure, which means that empathy components were not associated with current religious beliefs in our schizophrenia patients. 

In relation to childhood religious practice (Table S2), both age (β = 0.22; *p* = 0.033) and female gender (β = 0.39; *p* < 0.001) were significantly associated with this religiousness dimension. Positive and negative symptoms were not associated with childhood religious practice. Of all IRI dimensions, only perspective-taking entered the final equation, and was positively associated with childhood religious practice (β = 0.25 *p* = 0.019).

In relation to moral relativism (Table S3), age, female gender, and positive and negative symptoms were not significantly associated with this religiousness dimension. In a similar way to that of the previous dimension, perspective-taking was the only IRI dimension that was significantly associated with moral relativism (β = 0.33; *p* = 0.008).

### 3.3. Multiple Linear Regression Analyses in the Control Group

#### 3.3.1. Overall Religiousness

We also conducted a multiple linear regression in the control group to address whether IRI dimensions were associated with religiosity (Table S4, Supplementary Results). None of the IRI dimensions were associated with religiosity (total score) in the general population. In the final equation in the control group, age was associated with religiosity (β = 0.20; *p* = 0.025), but female gender was not.

#### 3.3.2. Religiousness Dimensions

In relation to current religious beliefs (Table S5, Supp. Results), age and female gender were not associated with this religiousness dimension. None of the IRI dimensions were associated with this dimension. 

In relation to childhood religious practice (Table S6, Supp. Results), age was significantly associated with this religiousness dimension (β = 0.24; *p* = 0.006). None of the IRI dimensions were associated with this dimension. 

In relation to moral relativism (Table S7, Supp. Results), female gender was significantly and inversely associated with this religiousness dimension (β = −0.28; *p* = 0.001), which means that women from the control group reported less moral relativism than men. Of all IRI dimensions, perspective-taking (β = 0.30; *p* < 0.001) was associated with moral relativism.

## 4. Discussion

Our stable schizophrenic patients reported elevated religiosity scores compared to the control group, similarly to other samples [1,3,4]. Regarding empathy measures, these patients showed differences in the three components of the interpersonal reactivity index (IRI): Lower perspective-taking and emphatic concern, as well as more personal distress than the comparison group (Table 1), thus agreeing with previous findings using similar measures [21,22,27,28,42]. These findings, however, did not provide robust enough evidence to sustain the conjecture of a singular deficit in affective empathy in schizophrenia [24,26,44,45]. Systematic reviews and meta-analyses have summarized the deficits in performance-based tasks of theory of mind in patients with schizophrenia [46,47,48]. 

Concurring with all this, our findings highlighted the difficulties that schizophrenia patients might have in processing the emotional and cognitive states of others while experiencing more personal distress when observing other people’s suffering. Personal distress, as measured by IRI, has been considered an index of self-oriented feelings of anxiety rather than other-oriented processes involved in empathy, and it might not represent affective empathy [19,26]. Bonfils et al. [22] insisted that high levels of personal distress in patients with schizophrenia may hinder their ability to empathically respond to others, and work against successful empathic interaction. Additionally, it is important to note that IRI was developed over three decades ago, and it was not originally intended to distinguish between cognitive and affective empathy. Its components may not clearly map onto the core cognitive and affective empathic processes [19]. 

The regression analyses depicted a positive relation between overall religiousness and the perspective-taking component of empathy after adjusting for significant variables linked to religiousness (age, female gender, and PANSS positive and negative syndromes) [2,3]. This result converges with other findings [13,16,17] that have suggested that differences in mentalistic abilities covariate with religiousness degrees. Mentalizing is the capacity of thinking about thinking and feeling; it gives the ability to selectively activate internal states while simulating mind states from other persons that might predict effects in the world. Mentalizing would create possibilities for individual interactions with God-images, which could induce effects on social functioning among schizophrenia patients [15,49]. Perhaps our patients, similarly to individuals with autism spectrum disorders, have distinctive processes of taking another’s perspective when incoming stimuli are only human-like rather than human [50], and can process them with fewer emotional risks. 

Furthermore, the regression analyses showed that positive symptom PANSS scores were associated positively with overall religiousness, while negative symptom PANSS scores were inversely related to it. Positive symptoms of psychosis largely involve illusory agents and experiences. The process of having vivid illusory experiences with social-like agents in the context of psychosis, and how they intrude, persist, and are experienced as psychologically credible, depends on alterations of neurocognitive systems dealing with social interactions [51,52,53]. Although auditory–verbal hallucinations have mainly been studied as individual phenomena [54,55,56], the most prominent illusory social experiences, delusional beliefs, may cause one to adopt implausible explanations and negative attributions about others [53]. Hallucinatory as well as delusional experiences may involve many forms, including those focused on supernatural agents or God-like figures [57]. In individuals experiencing fleeting psychotic-like symptoms in the absence of a formal psychotic disorder, elevated intrinsic religiosity was accompanied with volume reductions at the lateral and medial orbitofrontal cortex. Albeit not significant, an inverse link was found between negative psychotic-like symptoms and intrinsic religiosity [58].

No associations were found, finally, between overall religiosity and IRI dimensions of the control group of people from the community, thus confirming similar results to those obtained in other samples [36]. In these analyses, the strategy of progressively increasing the complexity of the models clearly identified the abovementioned effects in both size and direction. Once established, the greater overall religiosity within the schizophrenia patients and the potential links between psychotic symptoms and religiousness had to be assessed on patients only. Possible confusing effects of age, sex, and other variables also had to be assessed separately within the patient group. Patients’ ages were associated with religiousness (*p* = 0.09) when psychotic symptoms were introduced (models 2 and 3, with respect to model 1, Table 2); despite the weight of this variable, it was lighter than in healthy controls (*p* = 0.025, model Table S4). On the contrary, schizophrenic women were always “more religious” than schizophrenic men, an association that did not appear on the control’s religiosity scores (model Table S7).

The regression analyses highlighted in both the patient and control groups that empathic perspective-taking was related to moral relativism (non-religious pragmatism), thus suggesting that perspective-taking capacities might have a role in everyday moral judgments that are adjustable according to the circumstances [59]. Empathic perspective-taking combined with moral relativism could help one to accommodate to ambiguous ethical scenarios, maximizing benefits and minimizing dangers in challenging social contexts and encounters.

In summary, the present study adds useful information on the potential relevance of religiousness in patients with schizophrenia. Substantial differences appeared between schizophrenia patients and healthy controls regarding religiousness and empathy components. Higher religiosity but lower empathy with increased personal distress characterized our stable patients. The results indicated a distinctive association between religiosity and empathic perspective-taking only for patients. Significant positive relationships were found between religiosity with positive psychotic symptoms, but inversely with negative psychotic symptoms. Additionally, moral relativism was linked to empathic perspective-taking for both patients and normative controls. 

Several limitations of our exploratory study need to be addressed. First, the relatively small sample size may fail to identify real differences (type II error) due to a lack of statistical power. Second, our findings come from self-report measures only and lack necessary contrasts with other measures or with actual behavior in different settings that might better inform about both empathy and religiousness. Third, we did not get direct measures of cognitive abilities or mental task variabilities among these schizophrenia patients, although all of them understood the instructions very well and were able to deal successfully with their routine daily tasks, both at the Day Unit and at home. Finally, we included a control group using people recruited from the general population (the nearby community). This control group was not systematically interviewed by a psychiatrist to discharge a potential mental disorder. Thus, it cannot be considered a genuine “healthy control group”, as perhaps a minority of these subjects could suffer from mental health problems. However, we would expect a similar proportion of psychiatric disorders to that in the general population.

## Figures and Tables

**Table 1:** Socio-demographic, clinical data, religiousness, and interpersonal reactivity (IRI) profiles of the sample.

	Schizophrenia Group (n = 81)	Control Group (n = 95)	*p* Values
**Socio-demographic data**			
Male gender	55 (67.9%)	52 (54.7%)	*p* = 0.075
Age	37.1 (8.8)	39.9 (9.7)	*p* = 0.053
Education level > 15 years	15 (18.5%)	76 (80.8%)	*p* < 0.001
Marital Status, single	62 (77.5%)	16 (17.2%)	*p* < 0.001
Living with own family	13 (16.0%)	73 (77.6%)	*p* < 0.001
Employed	4 (5.0%)	82 (91.4%)	*p* < 0.001
**Clinical symptoms and treatment**			
PANSS scale			
Positive subscale	12.7 (5.2)		
Negative subscale	19.2 (8.5)		
General psychopathology subscale	32.9 (10.1)		
Treatment			
Typical antipsychotic	61 (75.3%)		
Atypical antipsychotic	5 (6.2%)		
Combined antipsychotics (typical + atypical)	15 (18.5%)		
**Religiousness and Interpersonal reactivity (IRI) measures**			
Religiousness (Kapogiannis scale)	82. 0 (30.6)	57.2 (25.5)	*p* < 0.001
Religion beliefs	60.9 (27.4)	37.6 (22.6)	*p* < 0.001
Childhood religious practice	11.2 (5.6)	9.7 (4.9)	*p* = 0.055
Moral relativism	9.8 (4.4)	9.9 (4.2)	*p* = 0.91
Interpersonal reactivity scale	90.2 (14.1)	90.6 (10.8)	*p* = 0.80
Perspective-taking	23.3 (4.2)	25.1 (4.2)	*p* = 0.008
Empathic concern	25.8 (4.6)	27.1 (3.4)	*p* = 0.039
Personal distress	20.9 (5.3)	17.7 (4.8)	*p* < 0.001

Data are presented as the mean (standard deviation) for continuous variables, and as N (%) for categorical variables. Abbreviations: PANSS = Positive and Negative Syndrome Scale.

**Table 2:** Results of the multiple regression exploring the relationship between overall religiousness and interpersonal reactivity (IRI) measures in patients with schizophrenia.

	Model 1 Adjusted for Age and Gender (R^2^ = 0.13)		Model 2 + Psychotic Symptoms (R^2^ = 0.28)		Model 3 + IRI Dimensions (R^2^ = 0.33)	
	β	*p* value	β	*p* value	β	*p* value
Age	0.16	0.156	0.18	0.09	0.18	0.09
Female gender	0.30	0.010	0.35	0.001	0.29	0.008
PANSS positive score			0.38	0.002	0.44	<0.001
PANSS negative score			−0.41	0.001	−0.40	0.001
Perspective-taking					0.24	0.030

Religiousness (overall score) was considered the dependent variable. Statistical *p* values for the changes in R^2^ were: 0.008 (Model 1), 0.001 (Model 2), and 0.030 (Model 3). Abbreviations: IRI = Interpersonal reactivity index; β = standardized beta coefficient; PANSS = Positive and Negative Syndrome Scale.

## Supplementary Materials

The following are available online at https://www.mdpi.com/2076-328X/10/2/53/s1, Table S1: Results of the multiple regression exploring the relationship between current religious beliefs and interpersonal reactivity (IRI) measures in patients with schizophrenia, Table S2: Results of the multiple regression exploring the relationship between childhood religious practice and interpersonal reactivity (IRI) measures in patients with schizophrenia, Table S3: Results of the multiple regression exploring the relationship between moral relativism and interpersonal reactivity (IRI) measures in patients with schizophrenia, Table S4: Results of the multiple regression exploring the relationship between religiousness and interpersonal reactivity (IRI) measures in the control group, Table S5: Results of the multiple regression exploring the relationship between current religious beliefs and interpersonal reactivity (IRI) measures in the control group, Table S6: Results of the multiple regression exploring the relationship between childhood religious practice and interpersonal reactivity (IRI) measures in the control group, Table S7: Results of the multiple regression exploring the relationship between moral relativism and interpersonal reactivity (IRI) measures in the control group.
