# Supplementary material for: Religiosity and Psychotic Ideation in Stable Schizophrenia: A Role for Empathic Perspective-Taking
Source: Behav Sci (Basel). 2020 Feb 5;10(2):53. doi: 10.3390/bs10020053 (PMC7071488; doi:10.3390/bs10020053)
Supplement: Supplementary file 1 [file behavsci-10-00053-s001.pdf]

SUPPLEMENTARY RESULTS

Table S1. Results of the multiple regression exploring the relationship between current religious beliefs and interpersonal reactivity (IRI) measures in patients with schizophrenia.

	Model 1 adjusted for age and gender (R^2= 0.09)		Model 2 + psychotic symptoms (R^2= 0.25)	
	β	p value	β	p value
Age	0.15	0.189	0.18	0.107
Female gender	0.22	0.057	0.28	0.012
PANSS positive score			0.40	0.002
PANSS negative score			-0.43	0.001

Current religious beliefs (Kapoggianis subscale) was considered the dependent variable. Statistical p values for the change in R^2 were: 0.045 (Model 1) and 0.001 (Model 2). None of the IRI dimensions entered the final equation. Therefore, only two models are shown. Abbreviations: IRI= Interpersonal reactivity index; β= standardized beta coefficient; PANSS= Positive and Negative Syndrome Scale.

Table S2. Results of the multiple regression exploring the relationship between childhood religious practice and interpersonal reactivity (IRI) measures in patients with schizophrenia.

	Model 1 adjusted for age and gender (R^2= 0.28)		Model 2 + psychotic symptoms (R^2= 0.29)		Model 3 + IRI dimensions (R^2= 0.34)	
	β	p value	β	p value	β	p value
Age	0.23	0.027	0.23	0.032	0.22	0.033
Female gender	0.44	<0.001	0.45	<0.001	0.39	<0.001
PANSS positive score			0.07	0.559	0.13	0.281
PANSS negative score			-0.06	0.620	-0.06	0.642
Perspective taking					0.25	0.019

Childhood religious practice (Kapogiannis subscale) was considered the dependent variable. Statistical p values for the change in R^2 were: <0.001 (Model 1), 0.818 (Model 2) and 0.019 (Model 3). Abbreviations: IRI= Interpersonal reactivity index; β= standardized beta coefficient; PANSS= Positive and Negative Syndrome Scale.

Table S3. Results of the multiple regression exploring the relationship between moral relativism and m and interpersonal reactivity (IRI) measures in patients with schizophrenia.

	Model 1 adjusted for age and gender (R^2= 0.02)		Model 2 + psychotic symptoms (R^2= 0.03)		Model 3 + IRI dimensions (R^2= 0.12)	
	β	p value	β	p value	β	p value
Age	-0.14	0.238	-0.13	0.285	-0.15	0.224
Female gender	0.07	0.544	0.08	0.513	-0.004	0.977
PANSS positive score			0.04	0.800	0.11	0.420
PANSS negative score			-0.07	0.636	-0.06	0.660
Perspective taking					0.33	0.008

Moral relativism (Kapogiannis subscale) was considered the dependent variable. Statistical p values for the change in R^2 were: 0.454 (Model 1), 0.893 (Model 2) and 0.008 (Model 3). Abbreviations: IRI= Interpersonal reactivity index; β= standardized beta coefficient; PANSS= Positive and Negative Syndrome Scale.

Table S4. Results of the multiple regression exploring the relationship between religiousness and interpersonal reactivity (IRI) measures in the control group.

	Model 1 adjusted for age and gender (R^2= 0.04)	
	β	p value
Age	-0.20	0.025
Female gender	-0.014	0.868

Religiousness (Kapogiannis scale total score) was considered the dependent variable. Statistical p values for the change in R^2 were: 0.078 (Model 1). None of the IRI dimensions entered the final equation. Therefore, only one model is shown. Abbreviations: IRI= Interpersonal reactivity index; β= standardized beta coefficient.

Table S5. Results of the multiple regression exploring the relationship between current religious beliefs and interpersonal reactivity (IRI) measures in the control group.

	Model 1 adjusted for age and gender (R^2= 0.03)	
	β	p value
Age	0.17	0.051
Female gender	0.008	0.928

Current religious beliefs (Kapogiannis subscale) was considered the dependent variable. Statistical p values for the change in R^2 were: 0.148 (Model 1). None of the IRI dimensions entered the final equation. Therefore, only one model is shown. Abbreviations: IRI= Interpersonal reactivity index; β= standardized beta coefficient.

Table S6. Results of the multiple regression exploring the relationship between childhood religious practice and interpersonal reactivity (IRI) measures in the control group.

	Model 1 adjusted for age and gender (R^2= 0.07)	
	β	p value
Age	0.24	0.006
Female gender	0.14	0.111

Childhood religious practice (Kapogiannis subscale) was considered the dependent variable. Statistical p values for the change in R^2 were: 0.007 (Model 1). None of the IRI dimensions entered the final equation. Therefore, only one model is shown. Abbreviations: IRI= Interpersonal reactivity index; β= standardized beta coefficient.

Table S7. Results of the multiple regression exploring the relationship between moral relativism and interpersonal reactivity (IRI) measures in the control group.

	Model 1 adjusted for age and gender (R^2= 0.09)		Model 2 + IRI dimensions (R^2= 0.18)	
	β	p value	β	p value
Age	0.01	0.953	0.01	0.876
Female gender	-0.30	<0.001	-0.28	0.001
Perspective taking			0.30	<0.001

Moral relativism (Kapogiannis subscale) was considered the dependent variable. Statistical p values for the change in R^2 were: 0.002 (Model 1) and <0.001 (Model 2). Abbreviations: IRI= Interpersonal reactivity index; β= standardized beta coefficient.

Further analysis involving other variables are available. Authors will provide them to interested readers on request,
